# Taxifolin as a Promising Ingredient of Cosmetics for Adult Skin

**DOI:** 10.3390/antiox10101625

**Published:** 2021-10-15

**Authors:** Iwona Micek, Joanna Nawrot, Agnieszka Seraszek-Jaros, Dorota Jenerowicz, Grzegorz Schroeder, Tomasz Spiżewski, Adela Suchan, Mariola Pawlaczyk, Justyna Gornowicz-Porowska

**Affiliations:** 1 Department and Division of Practical Cosmetology and Skin Diseases Prophylaxis, Poznan University of Medicinal Sciences, Mazowiecka 33, 60-623 Poznan, Poland; micekiwonax@gmail.com (I.M.); joannac@ump.edu.pl (J.N.); mariolapawlaczyk@o2.pl (M.P.); 2 Department of Bioinformatics and Computational Biology, Poznan University of Medical Sciences, 4 Rokietnicka Street, 60-806 Poznan, Poland; agnetpa@gmail.com; 3 Department of Dermatology, Poznan University of Medical Sciences, Przybyszewskiego 49, 60-356 Poznan, Poland; djenerowicz@ump.edu.pl; 4 Faculty of Chemistry, Adam Mickiewicz University in Poznan, Uniwersytetu Poznańskiego 8 Street, 61-614 Poznan, Poland; schroede@amu.edu.pl; 5 Department of Vegetable Crops, Poznan University of Life Sciences, Dąbrowskiego 159 Street, 60-594 Poznan, Poland; tomasz.spizewski@up.poznan.pl; 6 AVA Cosmetic Laboratory, Całowanie 103B, 05-480 Karczew, Poland; a.suchan@ava-laboratorium.pl

**Keywords:** taxifolin, *Stizolophus balsamita*, natural antioxidant, permeability, skin aging

## Abstract

Active substances, effective in the reduction in or delay of skin changes caused by aging occurring in natural compounds, are desirable. Taxifolin (TXF), a flavonoid of strong antioxidant activity found in the plant *Stizolophus balsamita* (*S. balsamita*), has been tested for its biological effects on adult human skin. The aim of the study was to investigate the effects of two creams: 3% *S. balsamita* extract and 3% TXF on the function of adult skin. In total, 97 Caucasian women with clinical signs of skin aging were investigated. The biophysical and biomechanical skin parameters were measured before and after applying the creams, using Colorimeter CL400, Mexameter MX16, Skin-pH-Meter PH900, Skin-Thermometer ST 500, Glossymeter GL200, and Cutiscan SC100. Patch tests were performed with the investigated products to assess their potential irritant properties. The percutaneous penetration of creams was examined with the use of electrospray ionization mass spectrometry (ESI-MS) and confocal Raman spectroscopy. The 3% *S. balsamita* extract cream reduced hyperpigmentation, erythema, and elevated pH. All the tested preparations were proven to be nonirritant. A higher penetration rate was revealed for the 3% TXF cream than for the 3% *S. balsamita* extract cream. A total of 3% TXF cream improved skin viscoelasticity. The obtained results suggested that *S. balsamita* extract and TXF may be considered as ingredients of skincare products for adults.

## 1. Introduction

Skin plays various essential functions, such as protection, modulation of the passage of water and electrolytes, excretion, absorption, thermoregulation, and hormone synthesis. All skin layers are involved in the barrier defense, whereas the role of epidermis and stratum corneum (SC) against environmental factors is the most important [1,2,3,4]. As an external part of the body, skin is a major candidate and target for damaging free radicals [4]. Antioxidant levels are shown to be greater in the deeper basal skin layers than in the more superficial. During the aging process and following cumulative exposure to ultraviolet radiation (UV), levels of antioxidants are significantly reduced, weakening the skin barrier function [5].

Reactive oxygen species (ROS) were reported to regulate melanogenesis, as they may activate tyrosinase, followed by increased melanin synthesis in human melanocytes [6]. Antioxidants are responsible for the redox balance in melanocytes by quenching ROS and reducing oxidative stress. Thus, the exogenous antioxidants may reduce hyperpigmentation or decrease melanogenesis [6].

It is well-documented that free radicals and ROS are involved in the aging process [7]. In the skin, about 1.5–5% of the consumed oxygen is converted into ROS [8], which is regarded as the main cause of intrinsic aging. Senescent, non-dividing cells are found in higher levels in aged skin [9]. The increased expression of enzymes implicated in the extracellular matrix degradation and matrix metalloproteinase in senescent fibroblasts may be triggered by ROS. An aged epidermis shows various skin barrier impairments [9]. Moreover, cellular senescence is associated with an imbalance between pro-inflammation and anti-inflammation, resulting in a chronic, low-grade, pro-inflammatory state (“inflammaging”) [10].

As several natural products exhibit promising skin anti-aging properties with the fortification of the skin barrier, these compounds might provide new active substances with the desired activity. In this context, flavonoids, known for their strong antioxidant effects, including ROS scavenging and metal-binding activities, might be a new potential ingredient in cosmetics for aging skin. Flavonoids such as epicatechin and rutin are powerful radical scavengers [11]. There are also other flavonoids, such as quercetin, myricetin, and quercetrin, which help inhibit the production of superoxide radicals [12].

Flavonoids likely exhibit their antioxidant effect through the inhibition of tyrosinase-catalyzed oxidation of L-dihydroxyphenylalanine (L-DOPA) and scavenging of free radicals [13]. Flavonoids are known for their anti-inflammatory [14], anticancer, antimicrobial, antiviral, anti-angiogenic, anti-malarial, neuroprotective, anti-allergic, and anti-proliferative activity [15]. Most of the biological activity of flavonoids is attributed to their redox-modulatory and protein-kinase-inhibitory properties [16]. In vivo, skin-penetration studies of flavonoids provide evidence that, after topical application, they can be absorbed from the skin surface and penetrate deeper skin layers [16].

One of the common flavanonols with potent antioxidant properties is taxifolin (TXF), also known as dihydroquercetin. The antioxidant and antiradical effects of TXF were proven by different in vitro bioanalytical antioxidant methods [17,18]. Scientific data show that TXF can act as an anti-inflammatory [19], hypopigmented, [20] and antitumor [21] agent, mitigate oxidative DNA damage [22] and prevent UV-induced skin carcinogenesis [23]. TXF shows relatively low toxicity, protects the skin from photoaging, and inhibits melanogenesis [20,24]. It is also one of the most potent flavonoids in the inhibition of interferon γ (IFNγ)-induced Intercellular Adhesion Molecule-1 (ICAM-1) protein, as well as mRNA expression in human keratinocytes. TXF pre-treatment also potently inhibited IFNγ-induced ICAM-1 expression in a reconstructed human skin equivalent, suggesting its therapeutic potential in pathological skin conditions related to increased cell adhesion and inflammation [16]. In vivo trials with experimental animals showed a beneficial TXF effect for chemically induced atopic dermatitis-like lesions [25] and chemically induced burns [26,27].

TXF is commonly found in onions [28], milk thistle [29], maritime bark [30], and Douglas fir bark [31] in aglycone and glycoside form. It is also the dominant compound of *Stizolophus balsamita* (*S. balsamita*) inflorescences, which was the subject of our previous investigations [32,33]. We have recently shown that *S. balsamita* extract, with TXF as the dominant flavonoid, might decrease transepidermal water loss (TEWL) and, in this way, fix the barrier function of the epidermis, which is its specific biological feature. We also confirmed the bio-safe nature of TXF to human fibroblasts [34].

Antioxidants have been proposed as functional ingredients for anti-aging preparations. The anti-aging properties of plant extracts such as *Polypodium leucotomos* [35], *Camellia sinensis* [36], *Ixora parviflora* [37], *Coffea arabica* [38] or *Labisia pumila* [39] in cosmetics are linked to their ability to decrease the skin damage caused by ROS. The activity of antioxidants may also be associated with the regulation of enzymes involved in the skin’s aging progression, such as elastase and collagenase [18].

To meet the expectations of consumers, the beauty industry searches for effective ingredients of natural origins [40]. From this perspective, the use of TXF as a lead compound for the design of new natural skincare products for adults seems to be promising. Due to the lack of data on the effectiveness of TXF on adult skin, the present study aimed to investigate the permeability of two different creams containing pure TXF and *S. balsamita* extract with TXF as a dominant compound, and to assess the effect of these cosmetics on the facial skin parameters of adult women.

## 2. Materials and Methods

The research with human subjects followed the recommended guidelines, as set out in the Declaration of Helsinki (1964) and associated amendments.

The study on human participants was approved by the local Bioethical Committee (Poznan University of Medical Sciences, no. 356/19, obtained 7 March 2019, Poznan, Poland), and written informed consent was obtained from all participants.

### 2.1. Materials

Inflorescences of *S. balsamita* (Lam.) K. Koch (Asteraceae) were collected from the Botanical Garden of the Department and Division of Practical Cosmetology and Skin Diseases Prophylaxis, University of Medical Sciences in Poznan (Poznan, Poland), in which the voucher specimens (voucher numbers: 55/2014) are deposited. Seeds of *S. balsamita* were provided by the Botanical Garden in Teheran (Teheran, Iran), which were gathered from the natural habitat in Iran. The plant was identified by our botanist based on the information from Flora Iranica [41] and Flora Europea [42]. The method of *S. balsamita* extraction and its phytochemical characterization was described in detail in our previous studies [32,34]. The pH value of *S. balsamita* extract was 4.5.

Two creams were composed in the Department and Division of Practical Cosmetology and Skin Diseases Prophylaxis, Poznan University of Medical Sciences (Poznan, Poland), with different active substances: a 3% dry ethanol extract of the *S. balsamita* containing TXF as a dominant compound in one cream, which was light green in color, and the second was white with commercially available TXF (3%), obtained from Cyherb (Ciyuan Biotech, Xi’an, China). A multi-component medium with a pH 5.5 Lekobaza^®^ Pharma Cosmetic base (Fagron, Kraków, Poland) was used as a cream base.

### 2.2. Study Groups

In total, 97 Caucasian women were included in the study: 60 for the examination of skin’s biophysical and biomechanical parameters, 35 for patch-testing, and two for the tape-stripping tests.

The biophysical and biomechanical skin parameters were measured in 60 adult females, aged 35–61 years (mean age 44.2), from which three subgroups were distinguished depending on the cream used: (i) 20 with *S. balsamita* extract (3%); (ii) 20 with TFX (3%); (iii) 20 with placebo (only cream base). All respondents demonstrated Fitzpatrick phototype II or III [43] and signs of skin aging such as facial wrinkles, sagging, dryness, or uneven color. The exclusion criteria included: pregnancy, breastfeeding, history of hypersensitivity to cosmetics, signs of skin infection, or inflammation. Further criteria related to skin treatments were the use of oral retinoids, dermal fillers, radiofrequency, micro-focused ultrasound, laser resurfacing (in the previous year); botulinum toxin injections (in the previous 8 months); chemical peels (in the previous 2 weeks). Subjects were asked to abstain from changing any facial skin products for at least 2 weeks and to avoid exposure to sun prior to the study. The volunteers stayed in the test room for at least 15 min before the measurements, so the skin could acclimatize to room conditions. To minimize measurement errors, the volunteers were asked not to apply any cosmetics in the tested area before the examination. The measurements were performed before and 30 days after the application of the investigational products.

Patch tests were performed in 35 individuals, including 17 healthy, adult volunteers (aged from 28 to 61, mean age 46.4) with no underlying skin or allergic diseases or skin lesions on the tested area, and 18 patients suffering from eczema (contact allergic or irritant) aged from 32 to 68 (mean age 52.5).

### 2.3. Methods

#### 2.3.1. Tape-Stripping Procedure with Electrospray Ionization Mass Spectrometry

To assess the transdermal delivery of creams with pure TXF and *S. balsamita* extract, the tape-stripping procedure was performed [44].

Two formulations were examined: (i) 26.00 mg/cm^2^ 3% TXF cream (0.78 mg/cm^2^ of TXF), (ii) 26.00 mg/cm^2^ 3% *S. balsamita* extract cream (0.24 mg/cm^2^ TXF).

All formulations were tested on the volar aspect of the forearm of each subject. Two 5 × 6 cm application sites were demarcated, and the amount of 3 mg/cm^2^ was applied onto the assigned skin area with a plastic disposable syringe. The creams were left in contact with the skin for 2 h before removal [45].

The 10 × 19 mm tape of 3M Scotch Crystal (3M Crystal Clear Tape Scotch, Maplewood, MN, United States) was utilized to sequentially tape-strip the SC of the exposed square skin sites (10 times). The thickness of the epidermis before and after the stripping was determined with ultrasonography (USG) 48 MHz DermaView USB2.0 System (DRAMINSKI SA, Olsztyn, Poland).

After removal, the 10 × 19 mm tapes were placed into the tube with 0.4 mL of methanol. Obtained methanol solutions were analyzed for TXF content with the electrospray ionization mass spectrometry (ESI-MS). ESI-MS and ESI-MSn spectra were recorded using an amaZon SL ion trap (Bruker, Bremen, Germany) equipped with an electrospray ion source in infusion mode. To establish the range of ESI-MS measurements that would permit qualitative and quantitative determination of TXF in methanol solutions, in the range of negative ions (*m/z* 303 analytical signals), a series of 6 standard methanol solutions of TXF was prepared, in which the concentration of TXF varied from 2.500 to 0.001 mg/mL.

The limit of detection (LOD) is the concentration of a substance below which the identity of the substance cannot be distinguished from analytical artifacts. The LOD was calculated according to the definition: LOD = mean blank value + 3 × standard deviation. The signal *m/z* analyte was three times higher than the noise level. The limit of quantification (LOQ) is the concentration of a substance, below which the concentration cannot be determined with acceptable accuracy. The LOQ value was calculated as LOQ = 3 × LOD [46,47].

#### 2.3.2. Confocal Raman Spectroscopy

Based on the tape-stripping results, TXF skin penetration was examined with Confocal Raman spectroscopy.

The examined formulation involved the 3% TXF cream.

Raman spectra and images were recorded with a WITec Confocal Raman Microscope (WITec alpha300 R, Ulm, Germany) with an electron-multiplying CCD (EMCCD) camera. For excitation, the frequency-doubled Nd: YAG laser (532 nm), which is a part of the instrument, is used with an output of 10 mW on the sample; the spectral resolution was about 3 cm^−1^. The spectra were collected through a 20× air objective and NA = 0.4. The measurements of the spectra were recorded at the following parameters: laser power, 10 mW; number of accumulation, 10; accumulation time of a single spectrum, 0.5 s. Raman spectra were collected in the range of 0–3600 cm^−1^ for 532 nm.

The skin-layer permeability analysis was performed on cross-sections through layers of skin after incubation with the samples. Human abdominal skin samples from healthy females were obtained during plastic surgery. The skin was prepared in a six-well plate with phosphate-buffered saline (PBS) to prevent tissue dehydration. Afterward, the skin was treated with TXF and incubated for 6 h at 37 °C (5% CO_2_), and placed on slides for Raman spectroscopy. All experiments were performed in three biological repetitions, each of them in three technical repeats [48].

#### 2.3.3. Patch Test

Patch tests were conducted to determine the in vivo biosafety of creams with 3%TXF and 3% *S. balsamita* extract.

In both the study group and the control group, patch tests (PT) were conducted at the Division of Allergic and Occupational Skin Diseases of the Department of Dermatology, Poznan University of Medical Sciences with the 3% TXF cream, and cream with 3% *S. balsamita* extract. PTs were applied using Finn Chambers mounted on the intact skin of the interscapular area for 48 h. PT readings were performed 48 and 72 h after application. The results were interpreted in accordance with the guidelines of the International Contact Dermatitis Research Group (ICDRG) [49].

#### 2.3.4. Biophysical and Biomechanical Skin Properties

The effect of the TXF and *S. balsamita* extract on the biophysical and biomechanical parameters of skin was found according to the guidelines for the assessment of skin properties in non-clinical settings [50,51] using non-invasive skin bioengineering techniques with the Courage-Khazaka instruments (Courage-Khazaka Electronic, Köln, Germany) MPA-9 and a CutiScan CS 100 (Courage-Khazaka Electronic, Köln, Germany).

The Mexameter^®^ MX 18 probe (Courage-Khazaka Electronic, Köln, Germany) emits three specific wavelengths of light (568 nm, 660 nm, 880 nm) and assesses erythema and melanin content. Measurement uncertainty: ±5%. The average of the three measurements is presented in arbitrary units (AU) from 0 to 999. The diameter of the instrument is 24 mm, the diameter of the measuring area is 5 mm (area 19.6 mm^2^) [52].

Colorimeter CL 400 (Courage-Khazaka Electronic, Köln, Germany): the probe emits white LED light (from 440 to 670 nm) that is arranged circularly to uniformly illuminate the skin [52,53,54]. The emitted light is scattered in all directions. Some parts pass through the layers of the skin and are spread out of the skin. The probe measures the light that is reflected. The raw data are processed with a special color matrix and expressed as an xyz value (tristimulus) that can be calculated to provide an L*a*b. The L* values determine the lightness and darkness of a color and correlate well with the lightness and darkness of skin color. The a* value measures cutaneous erythema and is impacted by melanin composition and cutaneous blood flow. The b* value reveals the individual’s constitutional pigmentation and ability to tan, specifically, the change in carotenoids, melanin synthesis, and oxidation after UV exposure. L* and b* parameters can be used for constitutive pigmentation classification according to the ITA°.

Thermometer ST500 (Courage-Khazaka Electronic, Köln, Germany) measures skin surface temperature (°C), and is based on the infrared technique.

The measurement of the skin pH with pHmeter is based on a combined electrode, where both glass H+ ion-sensitive electrode and an additional reference electrode are placed in one housing. It is connected to a probe handle containing the measurement electronics [55].

Glossymeter GL200 (Courage-Khazaka Electronic, Köln, Germany) expresses the portion of directly reflected light (gloss) and the diffusely scattered portion from the skin surface. The measurement of gloss is based on the reflection of light sent to the skin [55].

CutiScan CS 10 (Courage-Khazaka Electronic, Köln, Germany) is designed to provide information on elastic and viscoelastic properties, as well as skin anisotropy and directionality. This device contains a probe that uniquely combines mechanical force with imaging. It consists of a suction ring (14 mm diameter) that pulls the skin uniformly in all directions under constant negative pressure. During suction and recovery, a high-resolution CCD camera inside the probe monitors the displacement of each pixel using an optical flow algorithm in a video. An overall graph is generated from this video, including values of 0–360°, as well as other quantitative parameters [55]. Cutiscan was adjusted to a basic loading cycle at a constant pressure of 400 mbar, held for 3 s, and then released, followed by a relaxation period of approximately 3 s. The Cutiscan reported V1 (maximum distribution during suction) and V2 (return values), expressed in pixels, and V3 (*V2/V1*), expressed as a percentage [53,54]. The pre- and post-application variables obtained with Cutiscan at angles of 0°, 45°, 90°, and 135° were compared. As stated by Rosado et al., an analysis based on only four different angles is representative of the 360° angle trend. However, to quantify skin anisotropy, all measurements from 360° must be considered [55,56].

Measurements were carried out on precisely defined skin areas (Mexameter—Courage-Khazaka Electronic, Köln, Germany, Colorimeter—Courage-Khazaka Electronic, Köln, Germany, Glossymeter—Courage-Khazaka Electronic, Köln, Germany, pHmeter—Courage-Khazaka Electronic, Köln, Germany, Thermometer—Courage-Khazaka Electronic, Köln, Germany): left cheek, 2 cm below the orbit on the left side of the face in the inter-pupillary line. For Colorimeter and Mexameter measurements, a test area (3 × 3 cm) was marked on the skin. The probe was applied to the skin surface (1.54 cm^2^). For each cream, three individual measurements were taken at any timepoint using Mexameter and Colorimeter, and the mean value was used to calculate the results. Two areas were examined with Cutiscan: one at the border of the occurrence of crow’s feet on the cheekbone, and the other 1–2 cm below the corner of the mouth, to obtain repeatability in the measurements.

All measurements were carried out under controlled conditions at 22–25 °C and an average relative humidity of 52–58%.

#### 2.3.5. Statistical Analysis

Statistical analysis was based on Software Statistica PL 10.0 (StatSoft, Inc., Tulsa, OK, USA). All results were first verified by a normality test (Shapiro–Wilk test), which confirmed the compliance with the Gaussian curve. The repeated measure ANOVA test was performed to compare all results between groups and the results before and after treatment in three examined groups. When differences were found, Bonferroni’s test was used. The assumed statistical significance was *p* < 0.05.

## 3. Results

### 3.1. Tape-Stripping Procedure with Electrospray Ionization Mass Spectrometry

**Figure 1:**
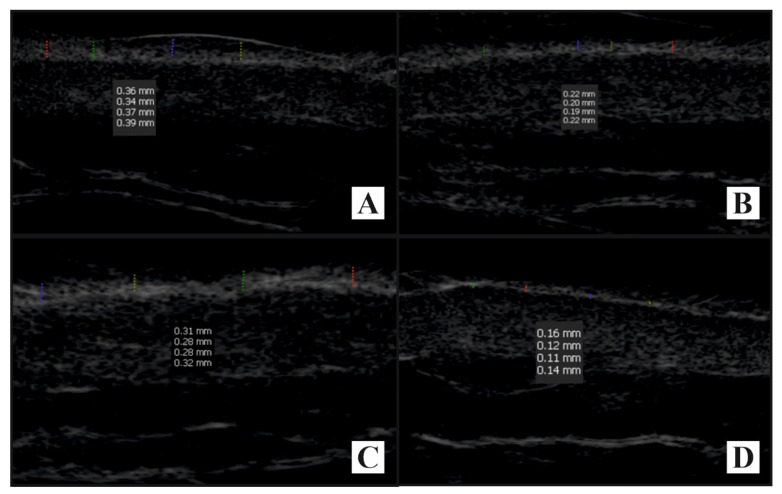
The epidermis thickness expressed in mm before ((**A**,**C**) mean value 0.37 mm and 0.30 mm, respectively) and after ((**B**,**D**) mean value 0.21 mm and 0.13 mm, respectively) stripping in two examined subjects.

The difference between epidermis thickness before and after 10 strippings was 0.17 mm, as shown in Figure 1.

The results of ESI-MS present a range of negative ions (*m*/*z* 303 analytical signal) for a series of six standard methanol solutions of prepared TXF, for which the concentration of TXF varied from 2.500 to 0.001 mg/mL, are demonstrated in Table 1.

Three external calibrations of TXF were used to validate the analytical method in terms of linearity, LOD, and LOQ. The limits of detection or quantification were good enough to appreciate the quantity of TXF present in the samples (LOD = 0.0005 ± 0.0002 mg/mL, LOQ = 0.0015 ± 0.0006 mg/mL, linear range = 0.001–2.500 mg/mL, correlation coefficient R^2^ = 0.998). The calibration dependence of determination of TXF using ESI-MS showed that the registered signal intensity corresponds to the concentration of TXF.

The amount of TXF in the first tape strip represented the amount remaining on the skin surface. Quantification of the nine following tape strips allowed for an assessment of the quantity of TXF that penetrated the stratum corneum layers from the topical application. A better penetration rate was revealed for the cream with 3% TXF than for the cream with 3% *S. balsamita* extract. After topical application, TXF from the 3% TXF cream showed a good diffusion profile through the first layers of the human SC. Detailed results are presented in Table 2.

### 3.2. Confocal Raman Spectroscopy

The cream with 3% TXF was investigated with confocal Raman spectroscopy (CRS), due to its higher penetration rate than the cream with 3% *S. balsamita* extract detected with the tape-stripping procedure.

**Figure 2:**
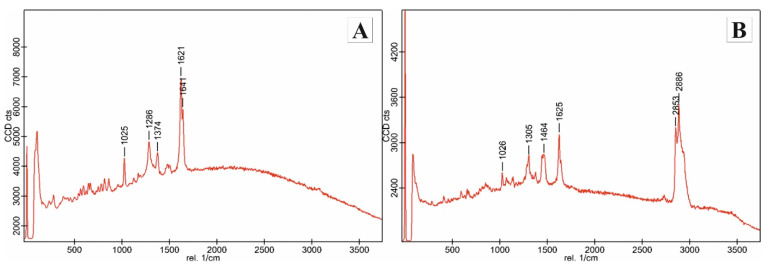
Raman spectra of taxifolin (**A**) and cream with 3% taxifolin (**B**).

The CRS spectra for TXF are provided in Figure 2.

**Figure 3:**
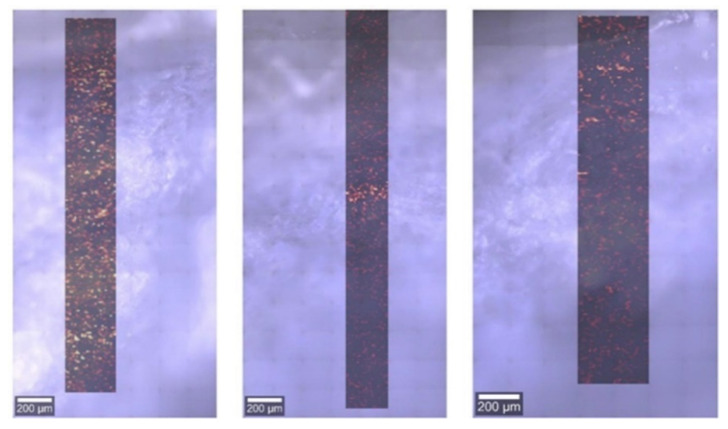
The representative Raman images for the cream with 3% taxifolin sample for each of Table 3. TXF was not observed for any of the recorded maps in cross-sections through the skin.

The representative Raman maps plotting the distribution of the cream with 3% TXF in the cross-section of the skin for a photomicrograph, taken in visible light, are presented in Figure 3.

### 3.3. Patch Testing

As presented in Table 3, no allergic or irritant reaction was observed.

The bands of cream with 3% TXF were not observed for any of the nine recorded maps in cross-sections through the skin.

### 3.4. Biophysical and Biomechanical Skin Properties

At the baseline, there were no differences between groups (*p* > 0.05). Detailed results of skin biophysical parameters (melanin index, erythema index, color L*a*b*, ITA°, pH, skin temperature, glossy) comparison, before and after treatment with examined creams, are presented in Table 4.

**Figure 4:**
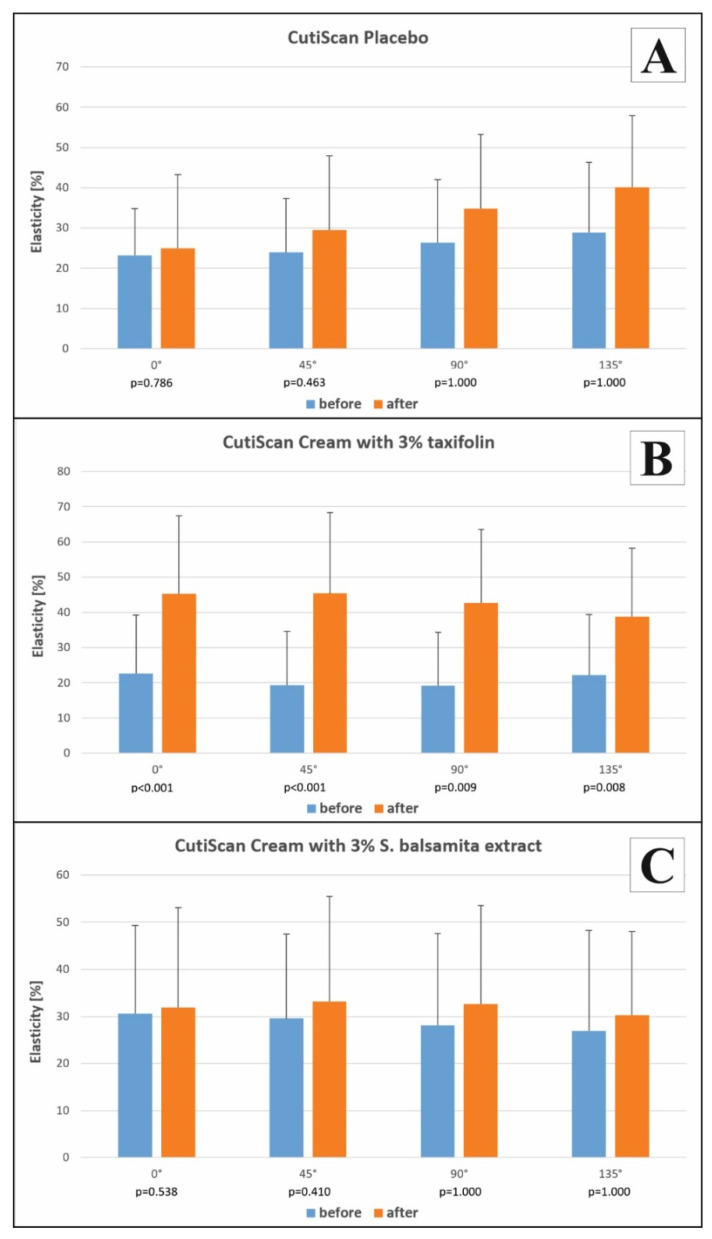
The comparison of skin viscoelasticity between the examined groups: placebo (**A**); cream with 3% taxifolin (**B**); cream with 3% *S. balsamita* extract (**C**). The results are expressed as mean ± standard deviation for angles 0°, 45°, 90°, and 135° before and after treatment. There was a significant difference between viscoelasticity measured before and after treatment for a cream with 3% taxifolin (*p* < 0.05) (**B**). There was no significant difference between viscoelasticity measured before and after treatment for placebo and cream with 3% *S. balsamita* extract (**A**,**C**). The statistical analysis for comparison between placebo and examined groups was as follows: placebo versus 3% TXF cream *p* = 0.001 for 0°, *p* = 0.010 for 45°, *p*= 1 for 90°, *p* = 1 for 135°; placebo versus 3% *S. balsamita* cream *p* = 0.386 for 0°, *p* = 0.725 for 45°, *p* = 1 for 90°, *p* = 1 for 135°.

There was a significant difference in the change rate of viscoelasticity improvement at both the cheek and canthus for the cream 3% TXF (*p*  <  0.05), as presented in Figure 4. There was no difference in the change rate of viscoelasticity improvement for the cream with 3% *S. balsamita* and placebo (*p* > 0.05).

## 4. Discussion

The skin, a protective organ, is the site of numerous biochemical processes, including the generation of free radicals (reactive oxygen and nitrogen species). Although ROS are necessary for biological signaling processes, oxidative stress is an integral element that plays a notable role in epidermal barrier disruption, skin aging, and melanogenesis. Exogenous triggers of oxidative stress, such as UV radiation and visible light, exert an influence on skin parameters through slightly different pathways, but they produce the same effect, generating skin barrier impairment and cosmetically undesirable hyperpigmentation [7,8,9].

In the present study, we extended our earlier research [32,34] on the interaction between the tested compounds and the skin barrier properties with non-invasive biophysical methods to measure skin color, melanin, erythema index, skin pH, skin-surface temperature, radiance, and viscoelasticity. First, we used the TEWL probe to measure water loss from the skin, reflecting the skin barrier’s condition through its water-retaining ability [34].

Our previous study [34] indicated that SC hydration and TEWL (*p* = 0.005), a marker of the inside–outside barrier, were improved after using the 3% *S. balsamita* extract cream. The TXF and placebo creams lacked similar observations. Thus, in the present work, we investigated the impact of treatment with *S. balsamita* extract and TXF on the biophysical (melanin index, erythema index, brightness, red intensity, yellow intensity, individual typological angle, pH, skin temperature, diffuse reflected/scattered light) and biomechanical (viscoelasticity) skin parameters, and related those effects to the skin penetration of the *S. balsamita* extract and TXF.

It is known that barrier abnormalities in the aged skin may be normalized by exogenously acidifying the SC [57]. Thus, acidification therapies with *S. balsamita* extract (pH = 4.5) may prevent the xerosis or eczema often observed in moderately aged women. According to previous clinical studies, a 4-week treatment with pH 4.0 skincare products significantly improved the SC integrity of the old population [58]. Therefore, the statistically significant reduction in the baseline skin surface pH after twice-daily applications of the cream with 3% extract of *S. balsamita* (*p* = 0.0323) could be due to the improvement in SC integrity. Interestingly, neither 3% TXF cream nor placebo reduced the skin pH.

Compounds that prevent and mitigate age-related inflammation and its effects have been an area of increasing interest in cosmetology. The utility of antioxidant therapy has been considered, given that melanogenesis is an oxidative process. Melanin is involved in the formation of skin barrier functions and exhibits antioxidant effects by scavenging free radicals [59]. Man et al. demonstrated that melanized keratinocytes displayed a superior barrier function compared to lightly pigmented keratinocytes [60]. It is suggested that skin with increased pigmentation may have different biophysical characteristics and, in particular, an altered skin barrier function [61]. Hyperpigmentation disorders, characterized by impaired stratum SC integrity, are major concerns in the human population. Moreover, age-related skin inflammation results in a compromised epidermal barrier, impaired moisture retention, erythema, scale, and pigment alteration [62]. The role of oxidative stress in melasma is well-defined, and it is documented that antioxidants possess clinical efficacy for the reduction in melanogenesis [63,64,65]. This is in line with our data, which suggest that TXF may be a new natural source of cosmetics, with therapeutic potential for patients with melasma. The measurements of MI indicated a significant reduction in melanin content for both creams, with 3% TXF and with 3% *S. balsamita* extract, but not for base cream (placebo). Additionally, skin tone was improved for the 3% *S. balsamita* extract cream but not for the 3% TXF cream and placebo. The findings obtained in the research on anti-inflammation compounds indicate that TXF and *S. balsamita* extract may reduce the objective signs of different phases of skin inflammation, such as hyperpigmentation and erythema.

Testing for the potential adverse skin effects (irritation and allergy) of novel compounds for cosmetics is essential. Thus, as a positive control group, we decided to test patients suffering from various types of eczema, prone to skin irritation and contact delayed-type hypersensitivity. The tested creams were shown to be nonirritant and non-allergenic, even for sensitive skin, confirming, at the in vivo level, our previous in vitro observations regarding the biosafety of TXF and *S. balsamita* extract.

Both 3% TXF and 3% *S. balsamita* extract creams significantly reduced skin temperature. Photoaging of the skin is characterized by a persistent, chronic inflammation that can increase temperature, similar to smoking or obesity [66,67,68]. Thus, this may suggest that both analyzed formulations are useful in skin-temperature regulation.

Improvements in the facial skin’s viscoelasticity [69] were only observed in TXF (*p* < 0.005), indicating that the 3% TXF cream successfully tensioned the skin, in contrast to the 3% *S. balsamita* extract cream and the placebo. However, it is possible that improvements in skin viscoelasticity may require a longer application time of the examined formulations, and further refinement of this parameter would be expected with continued use.

Variables such as penetrability can complicate the use of antioxidants. Thus, we decided to examine the transdermal delivery of 3% TXF cream and 3% *S. balsamita* extract cream. The data concerning the permeability of *S. balsamita* extract and TXF gave interesting results. The tape-stripping with ESI-MS indicated a higher penetration rate for the 3% TXF cream than for the 3% *S. balsamita* extract cream. This may be explained by the possibility of an existing bound form of TXF in *S. balsamita*, which may cause difficulties in skin penetration. The results obtained from the Raman mapping of the skin cross-section did not show the presence of the 3% TXF cream. This could be explained in two ways: (1) the sample did not penetrate the skin; however, in such a case, it should be seen on the skin surface, and this was not observed; (2) the cream penetrated the entire tested layer of skin, which is possible due to the quite long incubation period of the cream on the skin (6 h).

## 5. Conclusions

Our study confirms the beneficial effect of creams containing TXF on viscoelasticity and some skin biophysical parameters that change with age.

The obtained results suggested that the cream with 3% *S. balsamita* extract may be especially helpful in restoring the impaired epidermal barrier by acidifying and reducing hyperpigmentation and erythema. Its actions are likely associated with the synergistic interaction with other extract compounds, because TXF by itself does not generate similar results. The biological activity may also be associated with the occlusion provided by 3% *S. balsamita* extract cream, since only a slight permeation was demonstrated. Both creams proved to be biosafe.

## Figures and Tables

**Table 1:** ESI-MS results for a different concentration of taxifolin.

TXF Concentration (mg/mL)	Average Value	Signal Intensity *m/z* 302		
**2.500**	2.24 × 10^7^	2.22 × 10^7^	2.20 × 10^7^	2.29 × 10^7^
**1.568**	1.40 × 10^7^	1.40 × 10^7^	1.44 × 10^7^	1.37 × 10^7^
**0.941**	8.42 × 10^6^	8.09 × 10^6^	8.82 × 10^6^	8.36 × 10^6^
**0.272**	2.44 × 10^6^	2.35 × 10^6^	2.42 × 10^6^	2.55 × 10^6^
**0.092**	8.27 × 10^5^	8.28 × 10^5^	8.33 × 10^5^	8.19 × 10^5^
**0.001**	1.24 × 10^4^	1.10 × 10^4^	1.23 × 10^4^	1.40 × 10^4^

**Table 2:** The comparison of penetration between the cream with 3% *S. balsamita* extract and the cream with 3% taxifolin in subsequent layers of the stripped epidermis. cm^2^ stripped tapes [mg].

		Cream with 3% of Taxifolin (Quantity Applied: 26.00 mg/cm^2^)		Cream with 3% of *S. balsamita* Extract (Quantity Applied: 26.00 mg/cm^2^)	
No. of Layer	Thickness of Stripped Epidermis [mm]	Subject 1	Subject 2	Subject 1	Subject 2
1	0.017	0.323 ± 0.006	0.358 ± 0.006	0.416 ± 0.006	0.422 ± 0.006
2	0.034	0.295 ± 0.010	0.337 ± 0.027	0.016 ± 0.006	0.010 ± 0.006
3	0.051	0.284 ± 0.003	0.285 ± 0.010	0.014 ± 0.006	0.004 ± 0.006
4	0.068	0.270 ± 0.003	0.228 ± 0.006	0.014 ± 0.006	0.002 ± 0.006
5	0.085	0.225 ± 0.025	0.156 ± 0.003	0.005 ± 0.006	0.002 ± 0.006
6	0.102	0.089 ± 0.022	0.089 ± 0.001	0.002 ± 0.006	nd
7	0.119	0.032 ± 0.002	0.022 ± 0.001	nd	nd
8	0.136	nd	nd	nd	nd
9	0.153	nd	nd	nd	nd
10	0.170	nd	nd	nd	nd
Taxifolin quantity determined on the tapes		1.518 mg	1.475 mg	0.467 mg	0.440 mg

Abbreviation: nd—not determined (taxifolin content below the detection threshold).

**Table 3:** Results of patch testing with examined creams.

Suspected Cosmetics	Patients Tested			Positivity					
	Eczema Group (*n*)	Healthy Group (*n*)	Total (*n*)	Eczema Group (*n*)		Healthy Group (*n*)		Total (*n*)	
				After 48 h	After 72 h	After 48 h	After 72 h	After 48 h	After 72 h
**3% *S. balsamita* extract cream**	18	17	35	0	0	0	0	0	0
**3% TXF cream**	18	17	35	0	0	0	0	0	0

Descriptions: *n*—number of volunteers; h—hours; TXF—taxifolin.

**Table 4:** Detailed results of skin biophysical parameters comparison before and after treatment with the 3% *S. balsamita* extract cream and 3% TXF cream.

Parameter		3% *S. balsamita* Extraxt Cream (*n* = 20)	3% Taxifolin Cream (*n* = 20)	Placebo (*n* = 20)	Placebo Versus 3% *S. balsamita* Extract Cream	Placebo Versus 3% Taxifolin Cream (*p* Value)
**Melanin Index (AU)**	Before treatment Mean ± SD	120.0 ± 29.4	135.6 ± 22.1	125.1 ± 28.5		
	After treatment Mean ± SD	114.7 ± 30.7	129.0 ± 22.0	125.8 ± 36.3		
	*p* value	0.0618 *	0.0206 *	0.8728	0.3408	0.2012
**Erythema Index (AU)**	Before treatment Mean ± SD	372.9 ± 75.9	345.7 ± 75.0	349.6 ± 58.2		
	After treatment Mean ± SD	342.6 ± 72.0	337.6 ± 72.4	354.6 ± 77.8		
	*p* value	0.0069 *	0.3846	0.6407	0.0430 *	0.5117
Brightness L* (AU)	Before treatment Mean ± SD	62.8 ± 4.1	63.2 ± 3.3	63.9 ± 3.2		
	After treatment Mean ± SD	64.5 ± 3.3	64.0 ± 2.9	63.3 ± 3.2		
	*p* value	0.0028 *	0.2712	0.1167	0.0005 *	0.0402 *
Red intensity a* (AU)	Before treatment Mean ± SD	11.9 ± 1.9	11.1 ± 2.2	10.7 ± 1.5		
	After treatment Mean ± SD	10.9 ± 1.6	10.5 ± 1.9	10.9 ± 1.5		
	*p* value	0.0706 *	0.1459	0.1261	0.0283 *	0.9893
Yellow intensity b* (AU)	Before treatment Mean ± SD	11.1 ± 1.1	11.9 ± 1.9	11.4 ± 2.4		
	After treatment Mean ± SD	11.6 ± 1.5	11.3 ± 1.4	11.1 ± 2.4		
	*p* value	0.0034 *	0.1432	0.3898	0.0032 *	0.0375 *
**Individual typological angle ITA (°)**	Before treatment Mean ± SD	47.4 ± 11.4	46.7 ± 6.6	50.2 ± 8.6		
	After treatment Mean ± SD	50.5 ± 7.2	50.6 ± 4.9	49.8 ± 8.8		
	*p* value	0.0231 *	0.0111 *	0.7141	0.0167 *	0.0080 *
**pH**	Before treatment Mean ± SD	5.5 ± 0.4	5.4 ± 0.4	5.2 ± 0.5		
	After treatment Mean ± SD	5.3 ± 0.5	5.6 ± 0.3	5.5 ± 0.4		
	*p* value	0.0323 *	0.0352 *	0.0127 *	0.0014 *	0.6017
**Skin temperature in °C**	Before treatment Mean ± SDAfter treatment Mean ± SD	30.6 ± 1.729.6 ± 1.3	30.5 ± 1.329.8 ± 1.3	30.2 ± 1.230.0 ± 1.5		
	*p* value	0.0468 *	0.0314 *	0.5511	0.2766	0.3834
**Diffuse reflected/scattered light (AU)**	Before treatment Mean ± SD	25.3 ± 2.0	24.6 ± 3.0	26.0 ± 3.4		
	After treatment Mean ± SD	26.0 ± 2.8	26.0 ± 2.8	25.8 ± 3.4		
	*p* value	0.1432	0.0485 *	0.9019	0.4945	0.0460 *

Descriptions: *n*—number of volunteers; SD—standard deviation; AU—arbitrary unit; *—statistically significant for examined preparations before and after treatment.

## Data Availability

Data is contained within the article.
